# Clinical pharmacokinetics and pharmacodynamics of ivosidenib, an oral, targeted inhibitor of mutant IDH1, in patients with advanced solid tumors

**DOI:** 10.1007/s10637-019-00771-x

**Published:** 2019-04-26

**Authors:** Bin Fan, Ingo K. Mellinghoff, Patrick Y. Wen, Maeve A. Lowery, Lipika Goyal, William D. Tap, Shuchi S. Pandya, Erika Manyak, Liewen Jiang, Guowen Liu, Tara Nimkar, Camelia Gliser, Molly Prahl Judge, Sam Agresta, Hua Yang, David Dai

**Affiliations:** 1 DMPK, Agios Pharmaceuticals, Inc., 88 Sidney Street, Cambridge, MA 02139 USA; 2 Departments of Neurology and Human Oncology and Pathogenesis Program, Memorial Sloan Kettering Cancer Center, New York, NY USA; 3 Center for Neuro-Oncology, Dana-Farber Cancer Institute, Boston, MA USA; 4 Trinity St James Cancer Institute, Trinity College, Dublin, Ireland; 5 Department of Internal Medicine, Division of Hematology/Oncology, Massachusetts General Hospital Cancer Center, Boston, MA USA; 6 Department of Medicine, Memorial Sloan Kettering Cancer Center, New York, NY USA; 7 Department of Medicine, Weill Cornell Medical College, New York, NY USA; 8 Clinical Development, Agios Pharmaceuticals, Inc., Cambridge, MA USA; 9 Biostatistics - Clinical Development, Agios Pharmaceuticals, Inc., Cambridge, MA USA; 10 Clinical Operations - Clinical Development, Agios Pharmaceuticals, Inc., Cambridge, MA USA

**Keywords:** 2-hydroxyglutarate, Isocitrate dehydrogenase 1 inhibitor, Ivosidenib, Pharmacodynamics, Pharmacokinetics

## Abstract

*Background* Mutant isocitrate dehydrogenase 1 and 2 (IDH1/IDH2) enzymes produce the oncometabolite D-2-hydroxyglutarate (2-HG). Ivosidenib (AG-120) is a targeted mutant IDH1 inhibitor under evaluation in a phase 1 dose escalation and expansion study of IDH1-mutant advanced solid tumors including cholangiocarcinoma, chondrosarcoma, and glioma. We explored the pharmacokinetic (PK) and pharmacodynamic (PD) profiles of ivosidenib in these populations. *Methods* Ivosidenib was administered orally once (QD) or twice (BID) daily in continuous 28-day cycles; 168 patients received ≥1 dose within the range 100 mg BID to 1200 mg QD. PK and PD were assessed using validated liquid chromatography-tandem mass spectrometry assays. *Results* Ivosidenib demonstrated good oral exposure after single and multiple doses, was rapidly absorbed, and had a long terminal half-life (mean 40–102 h after single dose). Exposure increased less than dose proportionally. Steady state was reached by day 15, with moderate accumulation across all tumors (1.5- to 1.7-fold for area-under-the-curve at 500 mg QD). None of the intrinsic and extrinsic factors assessed affected ivosidenib exposure, including patient/disease characteristics and concomitant administration of weak CYP3A4 inhibitors/inducers. After multiple doses in patients with cholangiocarcinoma or chondrosarcoma, plasma 2-HG was reduced by up to 98%, to levels seen in healthy subjects. Exposure-response relationships for safety and efficacy outcomes were flat across the doses tested. *Conclusions* Ivosidenib demonstrated good oral exposure and a long half-life. Robust, persistent plasma 2-HG inhibition was observed in IDH1-mutant cholangiocarcinoma and chondrosarcoma. Ivosidenib 500 mg QD is an appropriate dose irrespective of various intrinsic and extrinsic factors. *Trial Registration*ClinicalTrials.gov (NCT02073994).

**Electronic supplementary material:**

The online version of this article (10.1007/s10637-019-00771-x) contains supplementary material, which is available to authorized users.

## Introduction

Isocitrate dehydrogenase (IDH) is a critical metabolic enzyme, catalyzing the oxidative decarboxylation of isocitrate to produce carbon dioxide and alpha-ketoglutarate (α-KG). Mutations in the *IDH1* and *IDH2* genes are found in multiple hematologic and solid tumors, including acute myeloid leukemia (AML) and glioma. Mutant IDH enzymes are not catalytically inactive, but rather possess a novel enzymatic activity, catalyzing the reduction of α-KG to the oncometabolite D-2-hydroxyglutarate (2-HG) [1, 2]. In normal cells, 2-HG is present at low levels. However, in cells with IDH1/IDH2 mutant enzymes, the accumulation of 2-HG alters a number of downstream cellular activities, causing epigenetic dysregulation and consequently a block in cellular differentiation, leading to tumorigenesis [3–5].

Ivosidenib (AG-120) is a selective, potent inhibitor of the mutant IDH1 protein [6]. Preclinical studies showed that treatment with ivosidenib decreased intracellular 2-HG levels in IDH1-mutant AML cells in vitro [7], and resulted in 2-HG inhibition in tumors in an IDH1-mutant xenograft mouse model [6]. These data were used to predict the exposure required for efficacy in humans. The inhibition of 2-HG production by ivosidenib translated well from preclinical models to humans [8]. In a phase 1 study, ivosidenib 500 mg once daily (QD) was shown to have an acceptable safety profile, and was associated with durable remissions in patients with advanced hematologic malignancies, including relapsed/refractory (R/R) AML and myelodysplastic syndrome [9]. On the basis of data from that study, ivosidenib received United States Food and Drug Administration (FDA) approval for the treatment of adult patients with R/R AML with a susceptible IDH1 mutation as detected by an FDA-approved test [10].

Ivosidenib is also being investigated in an ongoing phase 1 study that enrolled patients with advanced solid tumors [11–14]. The safety and efficacy data from this study are reported in separate publications (manuscripts in preparation). Here we report the pharmacokinetic (PK) and pharmacodynamic (PD) data associated with ivosidenib treatment in these patients, and the effects of intrinsic and extrinsic factors on ivosidenib clearance.

## Methods

### Study design and treatment

This was a phase 1, multicenter, open-label, dose escalation and expansion study (clinicaltrials.gov number NCT02073994). The primary objective was to assess the safety and tolerability of ivosidenib in patients with advanced solid tumors harboring an *IDH1* mutation. Secondary objectives included the characterization of ivosidenib PK and the PK/PD relationship of ivosidenib and 2-HG.

The study was conducted in accordance with the principles of the Declaration of Helsinki and Good Clinical Practice guidelines and was approved by the appropriate review boards at participating sites. Written informed consent was obtained from all patients.

In the dose escalation portion, patients with 1) glioma and 2) non-glioma solid tumors were enrolled into sequential cohorts using a standard 3 + 3 design. Patients with glioma received 100 mg twice daily (BID), or 300, 500, 600, or 900 mg QD ivosidenib in continuous 28-day cycles. Patients with cholangiocarcinoma, chondrosarcoma, and other solid tumors received ivosidenib 100 mg BID, or 300, 400, 500, 800, or 1200 mg QD in continuous 28-day cycles. At least 3 patients in each cohort also received a single dose 3 days prior to the start of multiple dosing (i.e., day −3). Patients in the expansion portion all received 500 mg QD ivosidenib in continuous 28-day cycles.

### Patients

All patients were required to be at least 18 years of age, and have an advanced solid tumor with an *IDH1* mutation, with an expected survival of at least 3 months, and adequate bone marrow, hepatic, and renal function. Other key inclusion criteria for dose escalation included histologically or cytologically confirmed advanced solid tumors that had recurred or progressed following standard therapy, and evaluable disease by Response Assessment in Neuro-Oncology (RANO) criteria for patients with glioma, or by Response Evaluation Criteria in Solid Tumors (RECIST) v1.1 for patients with other solid tumors. For expansion, patients with cholangiocarcinoma had to have a stage II, III, or IV intra-hepatic, extra-hepatic, or perihilar tumor that was not amenable to curative resection, transplantation, or ablative therapies (tumors of mixed histology were not allowed), and must have progressed following a gemcitabine-based regimen; patients with chondrosarcoma had to have a tumor that was either locally advanced or metastatic and not amenable to complete surgical excision (any subtype was permitted); patients with non-enhancing glioma had to have a tumor that had progressed within 12 months or less and was solely non-enhancing on magnetic resonance imaging (MRI), and have had no prior surgery or radiation therapy within 6 months of enrollment. Patients with other solid tumors in the expansion portion had to have a tumor that was refractory to conventional therapy or be unable to tolerate conventional therapy.

Key exclusion criteria for all patients included systemic anticancer therapy or radiotherapy less than 21 days prior to the first ivosidenib dose, use of investigational agents less than 14 days (or 5 half-lives) prior to the first dose, and concomitant use of sensitive substrates of cytochrome P450 (CYP) 3A4, or P-glycoprotein (P-gp).

### PK and PD assessments

For the first 3 patients enrolled in each cohort in the dose escalation portion, blood samples for assessment of plasma concentrations of ivosidenib and 2-HG were collected up to 72 h postdose on day −3. These samples were optional for any further patients enrolled in these cohorts. Further blood samples were collected from all patients up to 10 h postdose on cycle 1, day 15 and cycle 2, day 1. A subset of samples was also used for evaluation of plasma 4β-hydroxycholesterol (4β-OHC) and 4β-OHC:cholesterol ratios. Blood samples for assessment of 2-HG only were also collected at screening.

In the expansion portion, blood samples for assessment of plasma concentrations of ivosidenib and 2-HG were collected from all patients predose, and at 2, 3, 4, 6, and 8 h (±10 min) postdose on cycle 1, day 1 and cycle 2, day 1. Additional predose samples were taken on cycle 1, day 8; cycle 1, day 15; cycle 3, day 1; and end of treatment.

Tumor biopsies were also collected for 2-HG assessment at screening, and at day 1 of cycle 3. Further biopsies were collected depending on the status of disease at cycle 7, day 1 or at any time that disease progression was suspected and/or at the end of treatment.

Plasma ivosidenib was measured using two validated liquid chromatography-tandem mass spectrometry (LC-MS/MS) methods. The lower limit of quantification (LLOQ) was 1.00 ng/mL (low-range assay) or 50.0 ng/mL (high-range assay). The two methods were cross-validated and found to deliver comparable results with acceptable limits. Plasma 2-HG concentrations were measured using a qualified LC-MS/MS method with an LLOQ of 30.0 ng/mL. Concentrations of 2-HG in tumor biopsy samples were quantified using qualified LC-MS/MS methods with an LLOQ of 7.5 μg/g for brain tumor homogenate, 100 ng/g for liver tumor homogenate (low-range assay), and 30 μg/g for liver tumor homogenate (high-range assay). Plasma 4β-OHC and cholesterol were measured using a validated LC-MS/MS method. The LLOQ was 5 ng/mL for 4β-OHC and 25 μg/mL for cholesterol in human plasma.

Ivosidenib plasma PK parameters and 2-HG PD parameters were calculated using non-compartmental methods (Phoenix^®^ WinNonlin^®^ 6.3; Certara, Princeton, NJ). PK parameters included area under the plasma concentration-time curve (AUC), maximum plasma concentration (C_max_), time to maximum plasma concentration (T_max_), terminal elimination half-life (t_1/2_), and apparent oral clearance at steady state (CL_ss_/F). Accumulation ratio (R_acc_), based on C_max_ and AUC, was also assessed during the dose escalation portion. PD (2-HG) parameters included baseline-effect value, area under the effect concentration-time curve (AUEC_0-10h_ for the dose escalation portion, or AUEC_0-8h_ for the dose escalation and expansion portions), percent change from baseline in AUEC (%BAUEC_0-10h_ for the escalation portion, or %BAUEC_0-8h_ for the dose escalation and expansion portions), average plasma concentration (C_avg_), and percent inhibition for C_avg_ (%BC_avg_). For the dose escalation portion, the time-matched ratios of 4β-OHC to cholesterol were calculated. Box plots of 2-HG AUEC_0-8h_ and C_avg_ versus three categories of ivosidenib dose level (<500 mg, 500 mg and > 500 mg) were plotted at each timepoint to assess trends in the dose range studied (for dose escalation and expansion combined).

### Assessment of dose proportionality

Dose proportionality of ivosidenib AUC and C_max_ at day −3, and at cycle 1, day 15, and cycle 2, day 1 was assessed using a power model over the dose range studied (100 mg BID, and 300 mg to 1200 mg QD). Dose proportionality after multiple dosing was tested using data from the QD dose regimens without the 100 mg BID dose group. To assess dose proportionality, the confidence interval (CI) for the slope was estimated, with each natural logarithm (ln)-transformed PK parameter as the dependent variable and the ln-transformed dose as the fixed effect. Analysis was performed using PK parameter estimates at day −3, cycle 1, day 15, and cycle 2, day 1. Dose proportionality was concluded if the 95% CI around the slope included 1 and if the slope was between 0.8 and 1.2 (inclusive).

### Assessment of effects of intrinsic and extrinsic factors on ivosidenib PK

Patient characteristics at baseline were used to perform exploratory analyses of intrinsic and extrinsic factors that potentially influenced ivosidenib PK for two subgroups of tumor types: 1) non-glioma (cholangiocarcinoma, chondrosarcoma, and other solid tumors), and 2) glioma (non-enhancing and enhancing). Continuous factors assessed included age, body weight, body mass index, baseline creatinine clearance (CL_cr_), baseline estimated glomerular filtration rate (eGFR), and markers of hepatic function (alanine aminotransferase [ALT], alkaline phosphatase [ALP], aspartate aminotransferase [AST], bilirubin, total protein, and albumin). CL_cr_ was calculated using the Cockcroft and Gault equation and eGFR was calculated using the Modification of Diet in Renal Disease – National Kidney Disease Education Program equations [15]. Categorical factors assessed included sex, ethnicity, race, disease status, concomitant CYP3A4 inhibitor and inducer status, baseline hepatic and renal function category, and tumor type. Renal function was categorized as normal (CL_cr_ ≥ 90 mL/min or eGFR ≥90 mL/min/1.73 m^2^), mild impairment (CL_cr_ ≥ 60–89 mL/min or eGFR ≥60–89 mL/min/1.73 m^2^), moderate impairment (CL_cr_ ≥ 30–59 mL/min or eGFR ≥30–59 mL/min/1.73 m^2^), and severe impairment (CL_cr_ ≥ 15–29 mL/min or eGFR ≥15–29 mL/min/1.73 m^2^). Hepatic function category was based on National Cancer Institute (NCI) organ dysfunction working group (ODWG) criteria for hepatic dysfunction (0 = normal, 1 = mild, 2 = moderate, 3 = severe, 4 = liver transplant) [16].

### Exploratory dose/exposure-response analysis

A total of 6 adverse event categories, based on both laboratory abnormalities and reported adverse events, were included in the exposure-response analyses. Selected adverse events were identified based on safety signals observed within the ivosidenib clinical development program: grade ≥ 3 adverse events, serious adverse events, grade ≥ 2 gastrointestinal events (i.e., nausea/vomiting/diarrhea), hepatic enzyme elevations (i.e., newly occurring or worsening laboratory abnormalities of all grades, and grade ≥ 2 elevations in ALT, AST, or bilirubin), grade ≥ 2 decreases in lymphocytes, and grade ≥ 3 increases in ALP. The efficacy endpoint of interest was best response. The exposure-response analyses used modeled nominal steady-state C_max_ (C_max,ss_) or AUC (AUC_ss_) as predictors for safety and efficacy variables in the two subgroups of tumor types described earlier. The association between ivosidenib exposure and the selected endpoints was demonstrated by 1) exposure versus adverse event or clinical best response, with vertical boxplots showing exposure distributions, and 2) adverse event probability versus exposure plots showing the event probability for exposure quartiles and logistic regression fit based on the individual data.

## Results

### Patient disposition and demographics

The first patient was enrolled on March 14, 2014 and the study was ongoing at the time of writing. The data cutoff date for the analyses presented here was May 12, 2017. A total of 168 patients received at least 1 dose of ivosidenib, of whom 60 were treated in the dose escalation portion and 108 were treated in the expansion portion (Supplementary Fig. S1). The majority of patients were female (52.4%), white (78.6%), and < 60 years of age (68.5%). Tumor types included cholangiocarcinoma (43.5%), chondrosarcoma (12.5%), enhancing glioma (18.5%), non-enhancing glioma (20.8%), and other solid tumors (4.8%) (Supplementary Table S1). Renal function (based on CL_cr_) was classified as normal in 70.2% of patients, mild to moderate impairment in 29.2%, and severe impairment in 0.6%. Hepatic function (based on NCI-ODWG Classification) was classed as normal in 71.7% of patients, mild impairment in 26.4% and moderate impairment in 1.9%. Patients in the glioma cohort were generally younger than those in the other solid tumor subgroup, with a median age of 41 years (600 mg QD) and 35.5 years (900 mg QD) compared with 57 years (400 mg QD), 61 years (800 mg QD), and 55 years (1200 mg QD), respectively. PK and PD were assessed in all 168 patients treated with ivosidenib.

### PK

The single- and multiple-dose PK of ivosidenib are presented for patients with glioma (enhancing and non-enhancing) and non-glioma (cholangiocarcinoma, chondrosarcoma, and other solid tumors).

After a single dose on day −3, ivosidenib was rapidly absorbed, with median T_max_ ranging from approximately 2 to 6 h across all tumor types (Table 1). After reaching C_max_, mean ivosidenib concentrations declined in a bi-exponential manner over the 72-h postdose sampling period (Supplementary Fig. S2). The exposure of ivosidenib (C_max_ and AUC) generally increased in a less than dose-proportional manner after a single dose ranging from 100 to 1200 mg. At higher dose levels (500, 800, and 900 mg QD), mean AUC_0-72h_ and C_max_ in the subgroup of patients with glioma were lower than in the subgroup of patients with non-glioma solid tumors. Interpatient variability was low to moderately high for C_max_ and AUC parameters (i.e., geometric coefficient of variation [CV%] ranged from 7% to 66.9% where *n* ≥ 3). Mean t_1/2_ appeared to be shorter for the subgroup of patients with glioma (range ~40–56 h), versus the subgroup of patients with non-glioma solid tumors (range ~62–102 h).

**Table 1:** Summary of ivosidenib plasma PK parameters after a single oral dose of ivosidenib, by tumor-type subgroup (day −3, dose escalation)

PK parameter	Summary statistic^a^										
	Glioma					Non-glioma solid tumors					
	100 mg (*n* = 1)	300 mg (*n* = 6)	500 mg (*n* = 4)	600 mg (*n* = 5)	900 mg (*n* = 4)	100 mg (*n* = 3)	300 mg (*n* = 2)	400 mg (*n* = 5)	500 mg (*n* = 11)	800 mg (*n* = 6)	1200 mg (*n* = 5)
AUC_0-72h_ (ng•hr/mL)	32,835 (NC)	62,546 (18.7)	81,898 (24.2)	77,681 (42.5)	98,645 (24.2)^b^	34,825 (11.7)	71,555 (1.4)	140,607 (43.4)	112,490 (40.4)^f^	179,428 (35.0)	211,609 (52.9)
C_max_ (ng/mL)	1270 (NC)	2301 (14.4)	2374 (26.5)	3382 (23.6)	3436 (30.6)	1397 (35.6)	2352 (6.9)	4230 (7.0)	3657 (35.9)	6187 (36.5)	5989 (66.9)
T_max_ (hr)	2.03 (2.03; 2.03)	4.00 (2.08; 10.00)	3.54 (3.00; 8.00)	3.00 (2.85; 3.00)	2.55 (2.00; 6.00)	3.00 (1.50; 6.05)	4.17 (2.00; 6.33)	3.00 (0.98; 5.92)	2.92 (1.00; 4.02)	3.00 (1.00; 5.33)	6.00 (1.92; 7.88)
t_1/2_ (hr)	55.5 (NC)	53.6 (49.4)	56.4 (44.0)^b^	39.5 (40.0)	54.3 (55.3)^b^	102 (117.4)	77.9 (NC)^c^	77.3 (50.1)^d^	64.3 (25.3)^e^	62.8 (49.1)^f^	61.6 (69.4)

A subset of patients enrolled in dose escalation received a single dose of study drug on day −3 (i.e., only a single 100 mg dose for the 100 mg BID cohort). Glioma includes non-enhancing and enhancing glioma. Non-glioma includes cholangiocarcinoma, chondrosarcoma, and other solid tumors. Plasma ivosidenib LLOQ = 1.00 ng/mL or 50.0 ng/mL

*Abbreviations*: *AUC*_*0-72h*_ area under the plasma concentration-time curve from time 0 to 72 h postdose, *BID* twice daily, *C*_*max*_ maximum concentration, *LLOQ* lower limit of quantification, *NC* not calculated (*n* < 2), *t*_*1/2*_ apparent terminal elimination half-life, *T*_*max*_ time to maximum concentration

^a^Geometric mean (geometric coefficient of variation %) shown, except T_max_, which is median (minimum, maximum); ^b^*n* = 3; ^c^*n* = 1; ^d^*n* = 4; ^e^*n* = 10; ^f^*n* = 5

After multiple doses, ivosidenib was rapidly absorbed, and median T_max_ ranges (~2–3 h) were comparable in patients with glioma and patients with non-glioma solid tumors (Supplementary Tables S2 and S3). Mean concentration-time profiles of ivosidenib on cycle 1, day 15 were comparable with those on cycle 2, day 1 with QD dosing (data not shown), indicating that steady-state was reached within the first cycle. Exposure of ivosidenib (C_max_ and AUC) increased in a less than dose-proportional manner at doses from 300 to 1200 mg QD. At the 500 mg QD dose level, area under the curve over the dosing interval (AUC_0-tau_) and C_max_ in patients with glioma were lower than in patients with non-glioma solid tumors.

Mean CL_ss_/F generally increased with increasing dose and was slightly higher for patients with glioma (range 6–14 L/h) compared with patients with non-glioma solid tumors (range 3–10 L/h). Ivosidenib showed a consistent trend of moderate accumulation (1.5-fold to 1.7-fold for AUC at 500 mg QD) across tumor types (Supplementary Tables S2 and S3).

After multiple administrations of ivosidenib across the dose range from 100 mg BID and 300 mg QD to 1200 mg QD, mean plasma 4β-OHC:cholesterol ratios showed increases in the range of 86% to 258% from baseline to cycle 1, day 15 or cycle 2, day 1, and the increases did not appear to be dose dependent.

### Dose proportionality

**Fig. 1:**
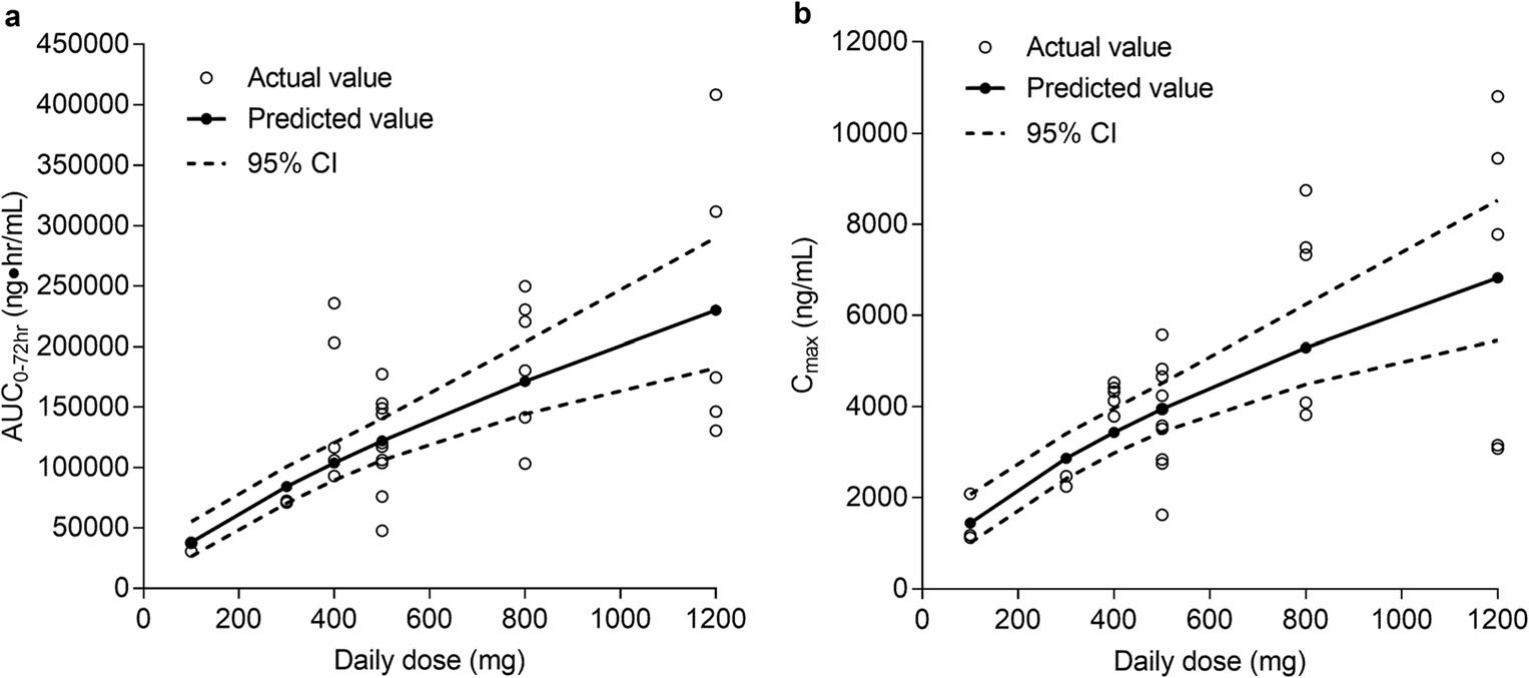
Representative graph of dose proportionality assessment for AUC_0-72h_ (**a**) and C_max_ (**b**) after single doses of ivosidenib (day −3) in patients with cholangiocarcinoma, chondrosarcoma, and other solid tumors (power model with 95% CI). Point-wise 95% CIs are shown. Ln-transformed PK parameters were back-transformed to the original scale by exponentiation

The point estimates and 95% CI for slope terms of the power models of AUC_0-72h_ and C_max_ after a single dose of ivosidenib on day −3 were 0.455 (95% CI 0.210–0.700) and 0.440 (95% CI 0.233–0.647), respectively, for glioma, and 0.726 (95% CI 0.513–0.938) and 0.625 (95% CI 0.419–0.831), respectively, for non-glioma solid tumors. Since the 95% CIs did not include 1 for any of the PK parameters, dose proportionality was not demonstrated over the dose ranges studied. Overall, the slope was less than 1, suggesting that C_max_ and AUC_0-72h_ exhibited less than dose-proportional increases in ivosidenib exposure in patients with glioma over the dose range of 100 mg to 900 mg, and in patients with non-glioma solid tumors over the dose range of 100 mg to 1200 mg (Fig. 1).

After multiple administrations of ivosidenib, at cycle 2, day 1, the point estimates and 95% CI for slope terms of the power models for AUC_0-tau_ and C_max_ were 0.249 (95% CI –0.109–0.606) and 0.227 (95% CI –0.179–0.634), respectively, for glioma, and 0.402 (95% CI 0.038–0.767) and 0.458 (95% CI 0.151–0.764), respectively, for non-glioma solid tumors. As described for the single-dose analysis above, the 95% CIs did not include 1 for any of the PK parameters, and the slope was less than 1, suggesting that C_max_ and AUC_0-tau_ at steady state exhibited less than dose-proportional increases in ivosidenib exposure in patients with glioma over the dose range of 300 mg to 900 mg, and in patients with non-glioma solid tumors over the dose range of 300 mg to 1200 mg.

### Effect of intrinsic and extrinsic factors on ivosidenib PK

Exploratory graphical analysis suggested that there was no trend between ivosidenib PK parameters and the continuous baseline characteristics of age, body weight, body mass index, markers of hepatic and renal function, use of CYP3A inducers/inhibitors, albumin, sex, ethnicity, race, tumor type, and performance status (data not shown).

**Fig. 2:**
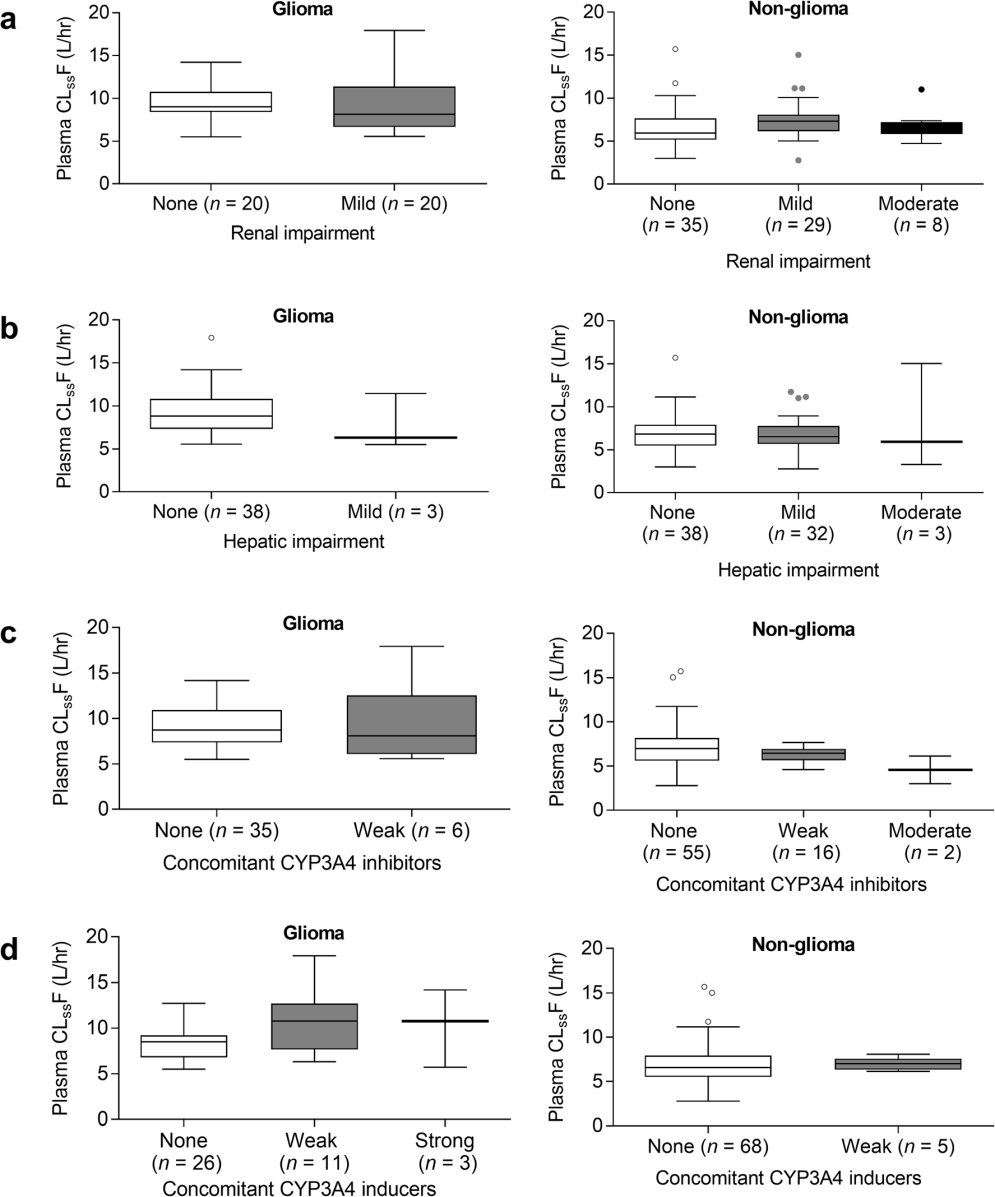
Plasma ivosidenib CLss/F after multiple oral doses of ivosidenib 500 mg QD (cycle 2, day 1, dose escalation and expansion) by renal function category (based on baseline eGFR) (**a**), hepatic function category (**b**), concomitant CYP3A4 inhibitors (**c**), and concomitant CYP3A4 inducers (**d**). Glioma includes non-enhancing and enhancing glioma. Non-glioma includes cholangiocarcinoma, chondrosarcoma, and other solid tumors. Horizontal lines denote median; boxes denote 25th to 75th percentiles; whiskers were plotted using the Tukey method

Exploratory assessments of categorical factors demonstrated that there was no apparent effect of mild or moderate renal impairment or mild hepatic impairment (Fig. 2a-b), or concomitant administration of weak CYP3A4 inhibitors or inducers (Fig. 2c-d) on ivosidenib CL_ss_/F. However, given the small sample size of patients with moderate hepatic impairment in the non-glioma solid tumor subgroup, the results of the comparison between moderate hepatic impairment and normal hepatic function should be interpreted with caution.

### Pharmacodynamics

In patients with glioma (non-enhancing and enhancing), mean plasma 2-HG baseline concentrations ranged from 49.7 ng/mL to 97.1 ng/mL and were similar to 2-HG levels previously observed in healthy subjects (72.6 ± 21.8 ng/mL; Agios data on file). Mean plasma 2-HG concentrations after ivosidenib treatment in patients with glioma remained similar to those observed in healthy subjects, therefore decreases from baseline in plasma 2-HG concentrations were not calculated, and this patient population was not investigated further with regard to PD parameters.

In patients with cholangiocarcinoma and chondrosarcoma, mean plasma 2-HG concentrations were elevated at baseline (ranging from 222 ng/mL to 5220 ng/mL and from 94 ng/mL to 1490 ng/mL, respectively) compared with healthy subjects. After one week of continuous ivosidenib dosing, plasma 2-HG was inhibited by up to 98% compared with baseline, to levels consistent with those seen in healthy subjects, and this decrease was maintained through the treatment period (up to 17 cycles of dosing).

### Correlative analysis of ivosidenib PK and PD

**Fig. 3:**
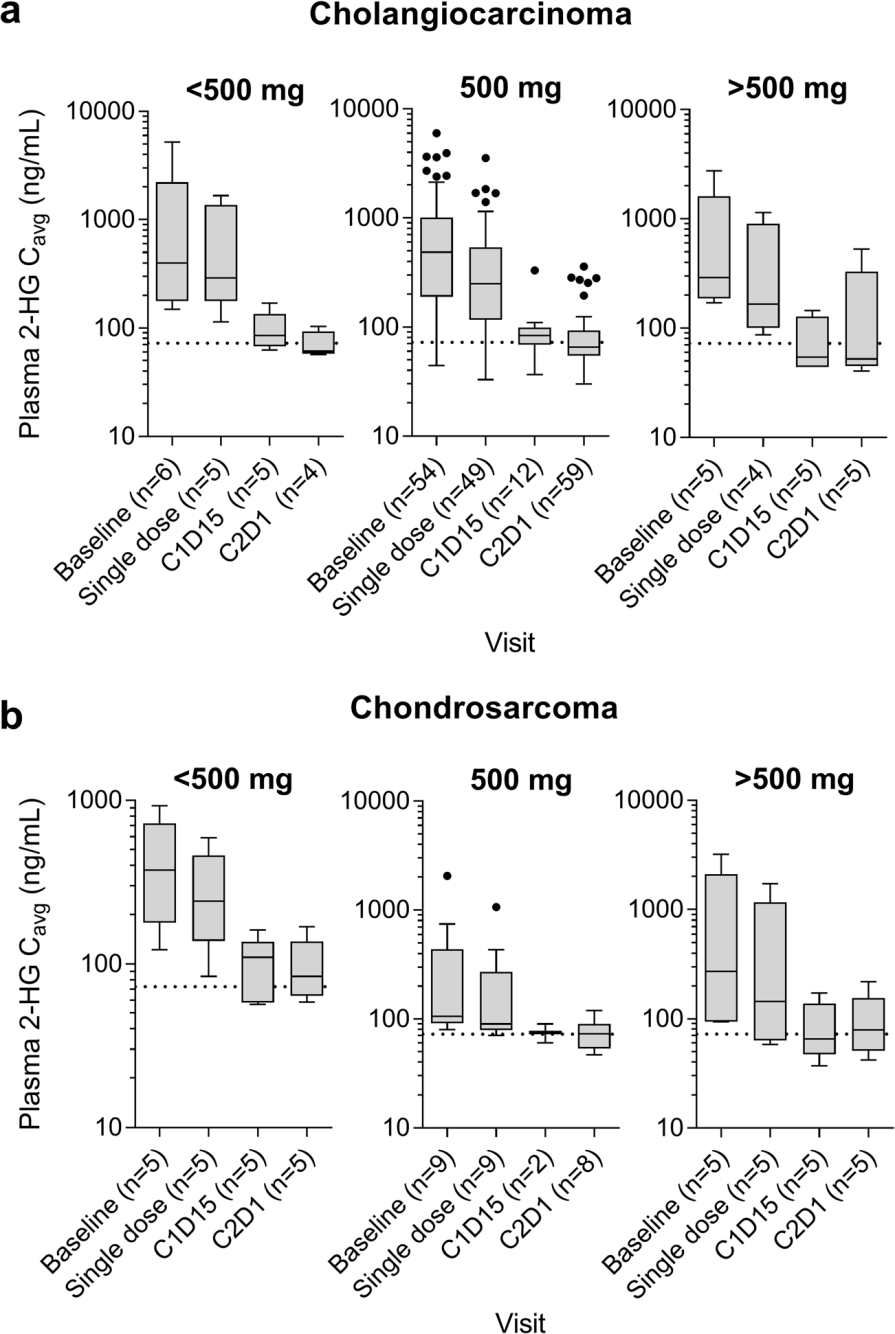
Summary of average plasma 2-HG concentrations over time by dose category and tumor type (dose escalation and expansion combined) for cholangiocarcinoma (**a**) and chondrosarcoma (**b**). The dotted horizontal lines denote average 2-HG levels observed in healthy volunteers. Abbreviations: C1D15, cycle 1 day 15; C2D1, cycle 2 day 1.

Following single-dose administration of ivosidenib, plasma 2-HG mean C_avg_ percent inhibition (BC_avg_%) was 30% and 46% in patients with chondrosarcoma and cholangiocarcinoma, respectively, for ivosidenib 500 mg QD. Plasma 2-HG mean BC_avg_% was higher following multiple-dose administration of ivosidenib in patients with chondrosarcoma and cholangiocarcinoma for doses of 500 mg: 54% and 79%, respectively, at cycle 1, day 15, and 59% and 80%, respectively at cycle 2, day 1. With the exception of ivosidenib doses below 500 mg, 2-HG inhibition (as assessed by plasma AUEC_0-8h_ and C_avg)_ following multiple-dose administration was observed as early as that seen following single-dose administration, with greater 2-HG inhibition after 15 days of QD dosing (cycle 1, day 15). The 2-HG inhibition at cycle 2, day 1 appeared to be similar to cycle 1, day 15 based on median plasma C_avg_ across all dose groups and tumor types (Fig. 3), indicating that steady-state plasma 2-HG inhibition was reached by cycle 1, day 15, and was maintained over the course of treatment. There was no additional 2-HG inhibition at doses >500 mg QD compared with doses of 500 mg QD.

**Fig. 4:**
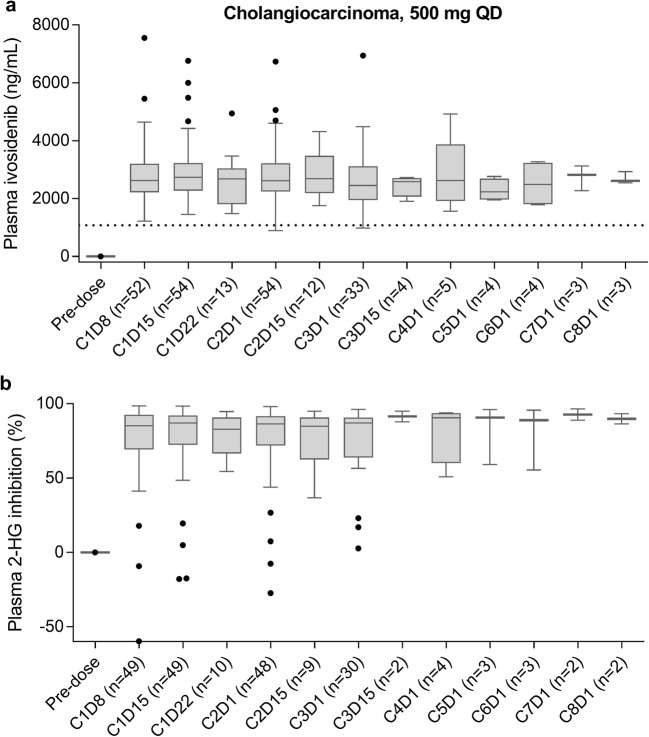
Longitudinal plots of plasma ivosidenib concentration (**a**) and percent 2-HG inhibition (**b**) after oral administration of ivosidenib 500 mg QD in patients with cholangiocarcinoma (dose escalation and expansion combined). The dotted horizontal line in panel a denotes the predicted efficacious level of ivosidenib

The longitudinal assessment of PK/PD revealed that plasma ivosidenib trough levels were maintained above the predicted efficacious exposure level (determined based on animal PK/PD studies, Agios unpublished data) throughout treatment, and 2-HG inhibition was not only robust but also persistent over the course of treatment. It was also confirmed that the steady state for ivosidenib plasma exposure and plasma 2-HG inhibition was reached within 14 days of cycle 1. A representative longitudinal PK/PD plot is shown in Fig. 4 for patients with cholangiocarcinoma receiving 500 mg QD.

Based on data from a limited number of paired tumor biopsy samples (21 patients; all tumor types included), multiple doses of ivosidenib 500 mg QD resulted in substantial 2-HG inhibition of up to 100% in tumors at cycle 3, day 1 and cycle 7, day 1 (Supplementary Fig. S3). Based on this limited sample set, exploratory correlations of 2-HG concentration in tumor biopsy samples versus 2-HG concentration in plasma suggested that plasma 2-HG concentration decreased with decreasing 2-HG concentration in tumors.

### Correlation of ivosidenib exposure with adverse events and clinical response

**Fig. 5:**
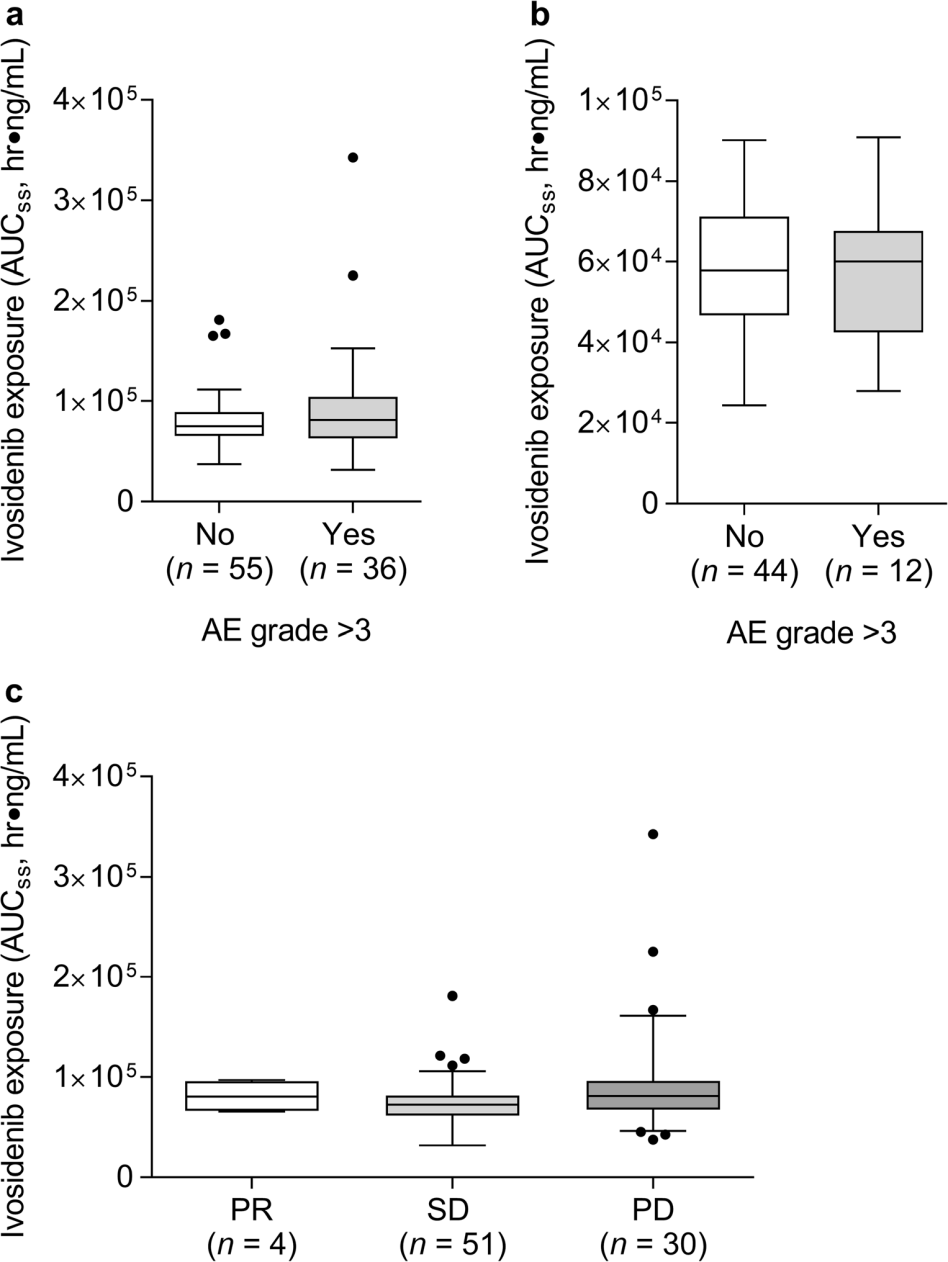
Exposure-response analyses. Ivosidenib exposure (AUC_ss_) distribution versus occurrence of grade ≥ 3 adverse events for patients with cholangiocarcinoma, chondrosarcoma and other solid tumors (**a**), and glioma (non-enhancing and enhancing) (**b**). Panel c shows exposure (AUC_ss_) distribution versus clinical best response in patients with cholangiocarcinoma, chondrosarcoma and other solid tumors. Abbreviations: AE, adverse event; PD, progressive disease; PR, partial response; SD, stable disease

Boxplots of exposure distributions (both C_max,ss_ and AUC_ss_) in patients who did and did not experience an adverse event in each category outlined in the methods section generally showed the exposure distributions to be similar and overlapping. A representative example of AUC_ss_ distribution in patients with or without grade ≥ 3 adverse events, split by tumor type, is presented in Fig. 5a-b. The observed adverse event incidence by exposure for AUC_ss_ or C_max,ss_ did not show clear trends for any of the adverse events assessed. Logistic regression analysis illustrated the absence of the effects of exposure (AUC_ss_) or C_max,ss_ on the incidence of all selected safety endpoints. Predicted changes over the exposure range were small and not clinically relevant.

Exposure distributions versus best clinical response were also generated. Both C_max,ss_ and AUC_ss_ distributions were similar and overlapping among patients with partial response, stable disease and progressive disease (Fig. 5c).

## Discussion

Ivosidenib demonstrated rapid oral absorption and was eliminated slowly, with a long half-life. Ivosidenib exposure increased with increasing dose; however, the increases were less than dose-proportional after both single and multiple doses. After multiple doses (cycle 2, day 1), AUC_0-tau_ and C_max_ increased approximately 6-fold and 4-fold, respectively, for a 12-fold increase in dose (100 mg BID to 1200 mg QD across all tumor types). A doubling of dose translates approximately to a 40% increase in ivosidenib exposure for patients with glioma and a 70% increase for patients with non-glioma solid tumors, across the dose range tested. The solubility, permeability, and dissolution characteristics of ivosidenib have been characterized according to the FDA Biopharmaceutical Classification System (BCS) guidance. Ivosidenib is considered to be a BCS Class II compound (i.e., low solubility, high permeability) (Agios data on file), and hence solubility-limited absorption may contribute to the non-proportionality observed after single and multiple dosing.

The estimated PK parameters for ivosidenib in patients with solid tumors (i.e., cholangiocarcinoma, chondrosarcoma, or glioma) exhibited time-dependency. Based on the mean t_1/2_ of 56 h and 64 h after a single dose of 500 mg in patients with glioma and cholangiocarcinoma/chondrosarcoma, respectively, the anticipated accumulation ratio would be approximately 4-fold after multiple dosing with 500 mg QD, whereas the observed accumulation ratios at cycle 2, day 1 were 1.5 and 1.7, respectively, suggesting an approximate 2.5-fold increase in CL/F after multiple dosing. Ivosidenib is mainly metabolized by CYP3A4, and induces CYP3A enzyme activity as suggested by increases in 4β-OHC:cholesterol ratios. On the basis of these data, it is plausible that autoinduction of ivosidenib metabolism may play a role in the observed increase in apparent clearance. However, the magnitude of autoinduction appears to be moderate, and approximately 1.5-fold accumulation was observed at steady state on day 15.

At the 500 mg QD dose level, AUC_0-24h_ and C_max_ in patients with glioma (enhancing and non-enhancing) were lower than in patients with cholangiocarcinoma/chondrosarcoma. A comparison at the recommended clinical dose of 500 mg QD indicates that the AUC_0-24h_ and C_max_ at steady state of ivosidenib are approximately 33% and 29% lower, respectively, in patients with glioma as compared with patients with cholangiocarcinoma/chondrosarcoma. Interindividual variability appears to be lower in patients with glioma than in patients with cholangiocarcinoma/chondrosarcoma. The lower exposure in glioma patients may reflect baseline patient-specific factors or disease characteristics in this population, such as lower age, less frequent use of CYP3A4 inhibitors, more frequent use of strong CYP3A4 inducers (e.g., carbamazepine and phenytoin), and adequate renal/hepatic function, as compared with patients with cholangiocarcinoma/chondrosarcoma.

The determination of the clinical dose of ivosidenib for the expansion portion of the study was based on PD (2-HG inhibition), PK, safety, and efficacy data from the dose escalation portion. Data from the combined dose escalation and expansion portions confirmed that the dose regimen of 500 mg QD ivosidenib appears appropriate for the treatment of patients with advanced solid tumors with an IDH1 mutation.

In patients with cholangiocarcinoma/chondrosarcoma, after multiple doses of ivosidenib, plasma 2-HG levels were substantially reduced (by up to 98%), to concentrations similar to those seen in healthy subjects, at all dose levels tested. No additional 2-HG inhibition was observed at doses >500 mg QD compared with 500 mg QD, while doses <500 mg QD appeared to be associated with lower levels of inhibition (although the sample size precluded statistical comparison). Continuous treatment with 500 mg QD was shown to provide persistent 2-HG inhibition, with no decrease in 2-HG inhibition over the whole treatment period. Unlike other solid tumor indications, plasma 2-HG does not appear to be a robust PD marker in the glioma population, which may be due to the anatomical location of the tumor. A perioperative study in patients with non-enhancing, IDH1-mutated, low-grade gliomas is being conducted to determine the concentration of ivosidenib and 2-HG in tumors following presurgical treatment with ivosidenib (clinicaltrials.gov NCT03343197). This study is ongoing and aims to confirm the optimal dose of ivosidenib in future glioma studies.

None of the intrinsic patient factors assessed, including renal and hepatic function, had an effect on ivosidenib exposure. Concomitant administration of weak CYP3A4 inhibitors or weak CYP3A4 inducers did not appear to affect the plasma clearance of ivosidenib. These results suggest that dose adjustment based on intrinsic patient factors or concomitant use of weak CYP3A4 inhibitors/inducers is not required.

The PK/PD analyses reported here indicate that ivosidenib has flat exposure-safety and exposure-efficacy relationships within the dose range tested. There was no observed effect of exposure on the incidence of any of the adverse events or safety endpoints assessed, and the exposure distribution was similar across all clinical response categories.

As reported elsewhere, ivosidenib has demonstrated encouraging preliminary clinical activity at a dose of 500 mg QD among the cholangiocarcinoma, chondrosarcoma, and non-enhancing glioma populations and is generally well tolerated, with an acceptable toxicity profile and few dose reductions or discontinuations owing to adverse events [11–14]. The data reported here demonstrate that ivosidenib has a favorable PK profile in patients with IDH1-mutated solid tumors, with robust suppression of plasma 2-HG in patients with cholangiocarcinoma or chondrosarcoma. The exposure-efficacy and exposure-safety results, combined with the consistency of the clinical benefit observed, suggest that ivosidenib 500 mg QD is an appropriate dose for the treatment of patients with IDH1-mutant advanced solid tumors.

## Electronic supplementary material

ESM 1 (PDF 237 kb)
