# Supplementary material for: Clinical pharmacokinetics and pharmacodynamics of ivosidenib, an oral, targeted inhibitor of mutant IDH1, in patients with advanced solid tumors
Source: Invest New Drugs. 2019 Apr 26;38(2):433–44. doi: 10.1007/s10637-019-00771-x (PMC7066280; doi:10.1007/s10637-019-00771-x)

Electronic Supplementary Material

Clinical Pharmacokinetics and Pharmacodynamics of Ivosidenib, an Oral, Targeted Inhibitor of Mutant IDH1, in Patients with Advanced Solid Tumors

Journal: Investigational New Drugs

Authors: Bin Fan, Ingo K. Mellinghoff, Patrick Y. Wen, Maeve A. Lowery, Lipika Goyal, William D. Tap, Shuchi S. Pandya, Erika Manyak, Liewen Jiang, Guowen Liu, Tara Nimkar, Camelia Gliser, Molly Prahl Judge, Sam Agresta, Hua Yang, and David Dai

Corresponding Author: Bin Fan, PhD, Agios Pharmaceuticals, Inc., 88 Sidney Street, Cambridge, MA 02139. E-mail: bin.fan@agios.com

Supplementary Table S1 Patient demographics and baseline characteristics

	Ivosidenib dose regimen								
**Parameter**	**100 mg BID (*n* = 4)**	**300 mg QD (*n* = 9)**	**400 mg QD (*n* = 5)**	**500 mg QD (*n* = 130)**	**600 mg QD (*n* = 5)**	**800 mg QD (*n* = 6)**	**900 mg QD (*n* = 4)**	**1,200 mg QD (*n* = 5)**	**Overall (*N* = 168)**
Sex *n* (%)									
Female	2 (50.0)	3 (33.3)	4 (80.0)	70 (53.8)	1 (20.0)	3 (50.0)	1 (25.0)	4 (80.0)	88 (52.4)
Male	2 (50.0)	6 (66.7)	1 (20.0)	60 (46.2)	4 (80.0)	3 (50.0)	3 (75.0)	1 (20.0)	80 (47.6)
Age years, mean (SD)	64.5 (18.57)	53.0 (15.55)	59.6 (7.83)	52.1 (13.25)	45.4 (12.18)	54.8 (15.07)	33.5 (6.14)	55.2 (10.89)	52.2 (13.58)
Race *n* (%)									
White	4 (100.0)	8 (88.9)	5 (100.0)	97 (74.6)	4 (80.0)	5 (83.3)	4 (100.0)	5 (100.0)	132 (78.6)
Black or African American	0	0	0	2 (1.5)	0	0	0	0	2 (1.2)
Asian	0	0	0	2 (1.5)	0	0	0	0	2 (1.2)
Other	0	0	0	2 (1.5)	0	0	0	0	2 (1.2)
Not Reported	0	1 (11.1)	0	27 (20.8)	1 (20.0)	1 (16.7)	0	0	30 (17.9)
Ethnicity *n* (%)									
Hispanic or Latino	1 (25.0)	2 (22.2)	0	5 (3.8)	0	0	0	0	8 (4.8)
Not Hispanic or Latino	2 (50.0)	6 (66.7)	5 (100.0)	90 (69.2)	4 (80.0)	5 (83.3)	4 (100.0)	5 (100.0)	121 (72.0)
Not reported	1 (25.0)	1 (11.1)	0	35 (26.9)	1 (20.0)	1 (16.7)	0	0	39 (23.2)
Baseline BMI in kg/m^2, mean (SD)	28.64 (4.905)	28.56 (4.035)	25.86 (2.674)	26.90 (5.602)	25.01 (4.956)	23.09 (3.743)	26.02 (0.748)	24.06 (7.590)	26.70 (5.395)

Disease type, *n* (%)									
Cholangio-carcinoma	2 (50.0)	3 (33.3)	1 (20.0)	62 (47.7)	0	2 (33.3)	0	3 (60.0)	73 (43.5)
Chondro-sarcoma	1 (25.0)	0	4 (80.0)	11 (8.5)	0	3 (50.0)	0	2 (40.0)	21 (12.5)
Enhancing glioma	1 (25.0)	4 (44.4)	0	22 (16.9)	4 (80.0)	0	0	0	31 (18.5)
Non-enhancing glioma	0	2 (22.2)	0	28 (21.5)	1 (20.0)	0	4 (100.0)	0	35 (20.8)
Other solid tumor	0	0	0	7 (5.4)	0	1 (16.7)	0	0	8 (4.8)

Abbreviations: BID, twice daily; QD, once daily; BMI, body mass index

Supplementary Table S2 Summary of ivosidenib plasma pharmacokinetic (PK) parameters after multiple oral doses of ivosidenib, by tumor type subgroup (cycle 2, day 1, dose escalation). Glioma includes non-enhancing and enhancing glioma. Non-glioma includes cholangiocarcinoma, chondrosarcoma, and other solid tumors. Ivosidenib plasma LLOQ = 1.00 ng/mL or 50.0 ng/mL

	**Summary statistic[a]**									
	**Glioma**				**Non-glioma solid tumors**					
**PK parameter**	**300 mg QD (*n* = 4)**	**500 mg QD (*n* = 4)**	**600 mg QD (*n* = 5)**	**900 mg QD (*n* = 4)**	**100 mg BID (*n* = 2)**	**300 mg QD (*n* = 3)**	**400 mg QD (*n* = 5)**	**500 mg QD (*n* = 18)**	**800 mg QD (*n* = 5)**	**1,200 mg QD (*n* = 5)**
$AUC_{0\text{-}10hr}$ (ng•hr/mL)	23,735 (33.5)	20,501 (44.3)	29,109 (14.0)	31,117 (17.5)	28,591 (21.3)	23,407 (37.9)[b]	51,554 (65.0)	33,865 (26.5)	44,496 (37.2)	57,727 (46.3)
$AUC_{0\text{-}tau}$ (ng•hr/mL)	50,750 (31.4)	44,393 (38.0)	59,760 (16.3)	64,479 (15.9)	33,571 (20.8)	53,211 (32.2)	111,195 (74.8)	72,767 (28.6)	93,115 (43.2)	119,847 (48.4)
C_{max} (ng/mL)	3,294 (39.0)	2,901 (46.5)	3,795 (13.0)	4,105 (21.0)	3,557 (14.2)	3,157 (24.2)	6,797 (49.4)	4,416 (26.2)	6,047 (29.2)	7,576 (39.0)
T_{max} (hr)	2.54 (1.95; 6.00)	2.00 (1.00; 9.88)	2.00 (2.00; 3.00)	2.52 (2.00; 3.98)	2.50 (2.00; 3.00)	2.05 (2.00; 4.00)	3.03 (1.95; 4.08)	2.96 (0.87; 6.17)	2.00 (1.00; 4.00)	2.92 (2.00; 8.15)
CL_{ss}/F (L/hr)	5.91 (31.4)	11.3 (38.0)	10.0 (16.3)	14.0 (15.9)	2.98 (20.8)	5.64 (32.2)	3.60 (74.8)	6.87 (28.6)	8.59 (43.2)	10.0 (48.4)
$R_{acc(AUC)}$	1.67 (30.0)	1.34 (31.1)	1.45 (19.9)	1.27 (30.2)	3.76 (65.3)	2.13 (19.3)[b]	1.87 (60.2)	1.53 (24.3)[c]	1.22 (49.2)	1.23 (28.9)
$R_{acc(Cmax)}$	1.41 (40.3)	1.22 (54.1)	1.12 (19.0)	1.19 (22.5)	2.28 (59.2)	1.47 (18.9)[b]	1.61 (57.2)	1.27 (19.8)[c]	1.02 (39.8)	1.27 (36.0)

Abbreviations: *$AUC_{0\text{-}10hr}$*, area under the plasma concentration-time curve from time 0 to 10 hours postdose; *$AUC_{0\text{-}tau}$*, area under the plasma concentration-time curve from time 0 to the end of the dosing interval; *C_{max}*, maximum concentration; *CL_{ss}/F*, steady-state apparent clearance after multiple doses; *LLOQ*, lower limit of quantification; *NA*, not

applicable ($n < 2$); $R_{acc(AUC)}$, accumulation ratio (based on AUC), calculated as $AUC_{0\text{-tau}}$ (cycle 1, day 15 or cycle 2, day 1)/$AUC_{0\text{-12hr}}$ or $AUC_{0\text{-24hr}}$ (day –3); $R_{acc(Cmax)}$, accumulation ratio (based on C_{max}), calculated as C_{max} (cycle 1, day 15 or cycle 2, day 1)/C_{max} (day –3); T_{max}, time to maximum concentration.

[a]Geometric mean (geometric coefficient of variation, %) except T_{max} which is median (minimum, maximum); [b]$n = 2$; [c]$n = 11$.

Supplementary Table S3 Summary of ivosidenib plasma PK parameters after single and multiple oral administrations of ivosidenib 500 mg QD, by tumor type (dose expansion). Ivosidenib plasma LLOQ = 1.00 ng/mL or 50.0 ng/mL

		Summary statistic[a]					
					Expansion solid tumors not otherwise eligible for the cholangiocarcinoma, chondrosarcoma, or non-enhancing glioma cohorts		
	**PK parameter**	**Expansion cholangio-carcinoma**	**Expansion chondro-sarcoma**	**Expansion non-enhancing glioma**	**Enhancing glioma**	**Other**	**Overall**
Cycle 1, day 1	n	45	9	24	22	4	104
	AUC_{0-8hr} (ng•hr/mL)	19,675 (42.8)[b]	22,910 (41.3)	14,873 (30.8)	16,020 (26.7)[h]	13,751 (44.2)	17,647 (39.7)[i]
	C_{max} (ng/mL)	3,665 (37.8)	4,259 (46.0)	2,788 (31.2)	3,142 (24.1)	2,292 (51.2)	3,314 (37.8)
	T_{max} (hr)	3.08 (1.82; 6.00)	2.98 (1.85; 4.17)	2.58 (1.92; 4.17)	3.01 (2.00; 8.08)	2.55 (2.00; 3.17)	3.00 (1.82; 8.08)
Cycle 2, day 1	n	46	7	23	15	3	94
	AUC_{0-8hr} (ng•hr/mL)	28,954 (32.2)	36,205 (27.9)[f]	22,210 (26.1)	24,377 (23.1)	25,068 (30.7)	26,633 (31.9)[j]
	AUC_{0-24hr} (ng•hr/mL)	75,569 (34.3)[c]	88,662 (32.1)	55,667 (29.1)[g]	62,596 (23.4)	62,225 (32.4)	68,516 (34.3)[k]
	C_{max} (ng/mL)	4,584 (29.0)	5,298 (25.4)	3,544 (26.2)	3,799 (27.3)	4,128 (43.0)	4,208 (30.7)
	T_{max} (hr)	2.03 (1.83; 6.15)	2.08 (1.98; 4.12)	3.05 (1.92; 5.58)	2.03 (1.83; 4.17)	1.98 (1.92; 3.05)	2.14 (1.83; 6.15)
	CL_{ss}/F (L/hr)	6.62 (34.3)[c]	5.64 (32.1)	8.98 (29.1)[g]	7.99 (23.4)	8.04 (32.4)	7.30 (34.3)[k]
	$R_{acc(AUC)}$	1.51 (33.3)[d]	1.70 (37.2)[f]	1.49 (29.2)	1.49 (28.6)	1.73 (23.5)	1.52 (31.0)[l]
	$R_{acc(Cmax)}$	1.28 (31.1)[e]	1.41 (26.8)	1.27 (27.1)	1.18 (24.9)	1.66 (20.9)	1.28 (28.7)[m]

Abbreviations: *AUC_{0-8hr}*, area under the plasma concentration-time curve from time 0 to 8 hours postdose; *AUC_{0-24hr}*, area under the plasma concentration-time curve from time 0 to 24 hours postdose; *CL_{ss}/F*, steady-state apparent clearance

after multiple doses; C_{max}, maximum concentration; *LLOQ*, lower limit of quantification; $R_{acc(AUC)}$, accumulation ratio (based on AUC), calculated as $AUC_{0\text{-}tau}$ (cycle 2, day 1)/$AUC_{0\text{-}12hr}$ or $AUC_{0\text{-}24hr}$ (cycle 1, day 1); $R_{acc(Cmax)}$, accumulation ratio (based on C_{max}), calculated as C_{max} (cycle 2, day 1)/C_{max} (cycle 1, day 1).

[a]Geometric mean (geometric coefficient of variation, %) except T_{max} which is median (minimum, maximum); [b]*n* = 44; [c]*n* = 45; [d]*n* = 41; [e]*n* = 42; [f]*n* = 6; [g]*n* = 22; [h]*n* = 21; [i]*n* = 102; [j]*n* = 93; [k]*n* = 92; [l]*n* = 88; [m]*n* = 90.

Supplementary Fig. S1 Patient disposition (dose escalation and expansion combined)

Enrolled (*N* = 168)

Disposition	100 mg BID (*n* = 4)	300 mg QD (*n* = 9)	400 mg QD (*n* = 5)	500 mg QD (*n* = 130)[a]	600 mg QD (*n* = 5)	800 mg QD (*n* = 6)	900 mg QD (*n* = 4)	1,200 mg QD (*n* = 5)	Overall (*N* = 168)
**Treatment Status**									
On Treatment	0	0	0	35 (27%)	0	1 (17%)	3 (75%)	2 (40%)	41 (24%)
Discontinued Treatment	4 (100%)	9 (100%)	5 (100%)	95 (73%)	5 (100%)	5 (83%)	1 (25%)	3 (60%)	127 (76%)
**Primary Reason for Treatment Discontinuation**									
Progression of Disease	3 (75.0)	8 (89%)	3 (60%)	83 (64%)	5 (100%)	3 (50%)	1 (25%)	2 (40%)	108 (64%)
Clinical Progression	0	0	0	6 (4.6%)	0	2 (33%)	0	1 (20%)	9 (5.4%)
Adverse Event	0	1 (11%)	1 (20%)	1 (0.8%)	0	0	0	0	3 (1.8%)
Death	0	0	0	3 (2.3%)	0	0	0	0	3 (1.8%)
Withdrawal by Patient	1 (25.0)	0	1 (20%)	1 (0.8%)	0	0	0	0	3 (1.8%)
Lack of Clinical Benefit	0	0	0	1 (0.8%)	0	0	0	0	1 (0.6%)
**Patients Included in Analysis Data Sets**									
Full Analysis Set	4 (100%)	9 (100%)	5 (100%)	130 (100%)	5 (100%)	6 (100%)	4 (100%)	5 (100%)	168 (100%)
Safety Analysis Set	4 (100%)	9 (100%)	5 (100%)	130 (100%)	5 (100%)	6 (100%)	4 (100%)	5 (100%)	168 (100%)
PK Analysis Set	4 (100%)	9 (100%)	5 (100%)	130 (100%)	5 (100%)	6 (100%)	4 (100%)	5 (100%)	168 (100%)

[a]Included 22 patients in the dose escalation portion and 108 patients in the dose expansion portion

Abbreviations: *BID*, twice daily; *QD*, once daily

Supplementary Fig. S2 Mean (SD) ivosidenib plasma concentrations versus time after a single oral administration of ivosidenib, by tumor-type subgroup (day –3, dose escalation) for glioma (**a**) and non-glioma (**b**)

a

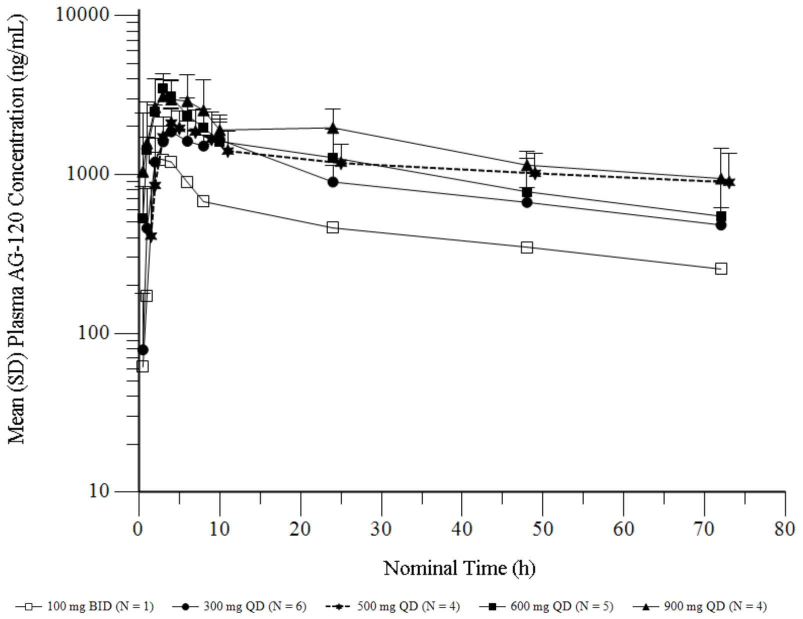

b

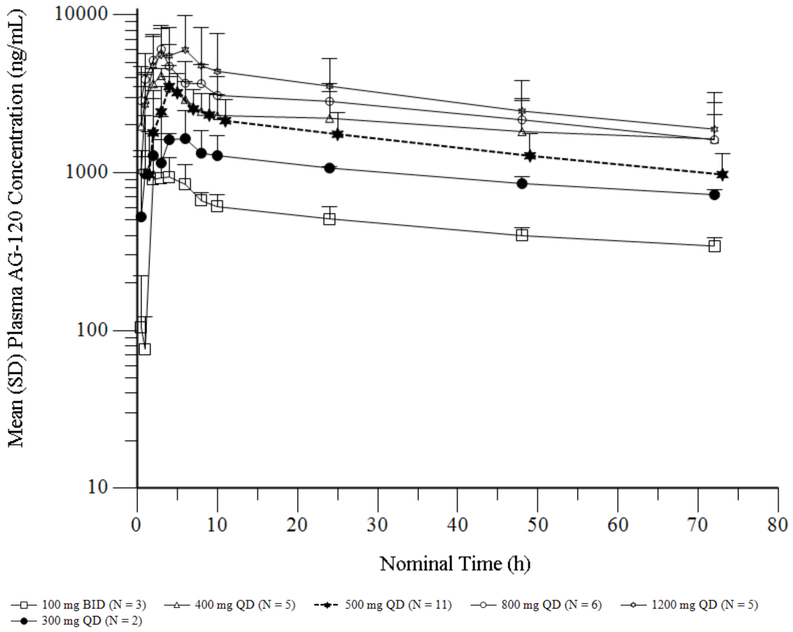

Supplementary Fig. S3 Tumor 2-HG concentration after ivosidenib administration by visit and dose group (dose escalation and expansion combined). All solid tumors are included but the majority of samples were cholangiocarcinoma or chondrosarcoma. P-values were calculated using a linear mixed effects model with dose group and visit as fixed effects and patient as random effect. For <500 mg QD and >500 mg QD, results should be interpreted with caution owing to very small sample size.

Abbreviations: *C3D1*, cycle 3, day 1; *C7D1*, cycle 7, day 1; *QD*, once daily

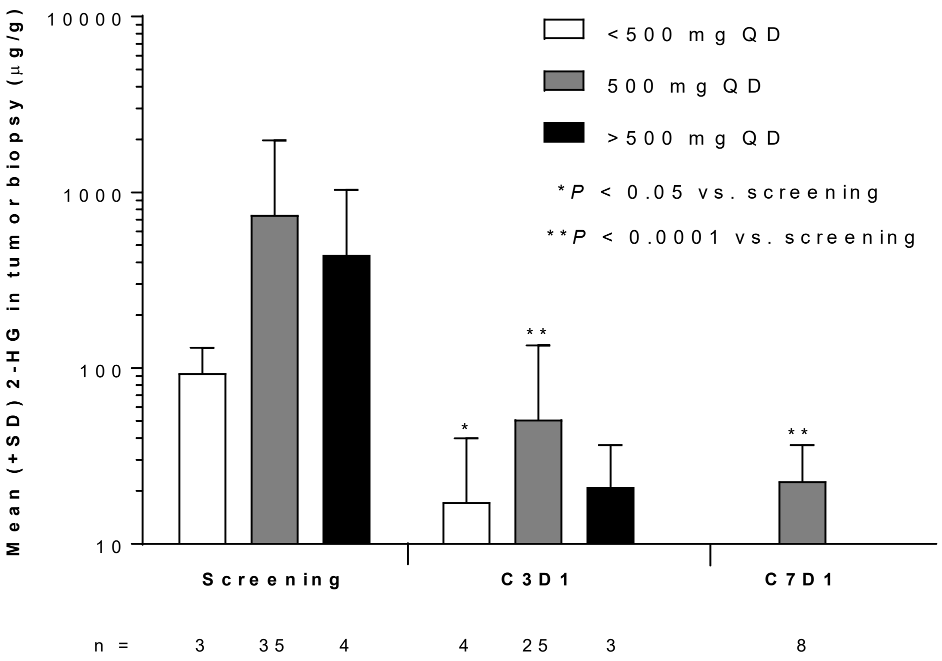

Supplement: Supplementary file 1 — (PDF 237 kb) [file 10637_2019_771_MOESM1_ESM.pdf]
